# Self-Compassion and Physical Activity: The Underpinning Role of Psychological Distress and Barrier Self-Efficacy

**DOI:** 10.3390/ijerph20021480

**Published:** 2023-01-13

**Authors:** Shuge Zhang, Clare Roscoe, Andy Pringle

**Affiliations:** School of Human Sciences, University of Derby, Kedleston Road, Derby DE22 1GB, UK

**Keywords:** self-compassion, psychological distress, barrier, self-efficacy, physical activity, sedentary, personality, disposition, self-regulation

## Abstract

Unlike other personality traits or dispositions, self-compassion can be nurtured and is likely a driving source for physical activity. Emerging research has started to examine self-compassion in physical activity contexts; however, most existing studies were underpowered and overlooked the psycho-behavioural factors underlying the link between self-compassion and physical activity. In a sample of 569 UK adults (mean age = 41.92 years, SD = 13.70; 47.8% female), we examined the hypothesis that self-compassion’s positive influence on physical activity operates through reduced psychological distress and subsequently increased barrier self-efficacy. Results supported the prediction, with the positive influences of self-compassion being more prominent in more vigorous physical activity. The findings suggest that self-compassion is a good source of emotional resources (i.e., attenuated psychological distress) and confidence to overcome challenges and obstacles (i.e., increased barrier self-efficacy) in the context of physical activity. Future interventions and programs could consider incorporating self-compassion for physical activity adoption and maintenance.

## 1. Introduction

Personality traits play a role in physical activity [1,2]. Emerging research also unveils that overlapping or shared genetic influences explain the phenotypic association between personality and physical activity among twin pairs [3]. The existing literature, therefore, suggests one’s personality characteristics serve as the predisposition for regular physical activity [4,5]. While such a proposition has received considerable support [6], most existing research in personality and physical activity predominately focused on the so-called Big-5 traits [7,8] and overlooked more specific manifestations of a personality beyond the Big-5 (for an exception, see [9]). Also, the Big-5, as with many personality traits, are relatively stable across the life span and considerably difficult to change or intervene with [10,11]. Therefore, one may see personality as more of a ‘moderator’ rather than a proximal ‘influencer’ of physical activity [12,13]. In this context, an important yet overlooked self-concept or dispositional characteristic that has the potential to be nurtured and can be a driving source for physical activity is self-compassion [14,15,16,17].

By definition [18], self-compassion involves the disposition of being touched by and open to one’s own suffering, not avoiding or disconnecting from it, generating the desire to alleviate one’s suffering and to heal oneself with kindness or acknowledging suffering as part of the normal, shared human experience. Besides, self-compassion is a healthy way of approaching one’s suffering, such as failures, perceived inadequacy, or other life difficulties. It allows an individual to accept distress or related feelings/experiences with a gentler mindset and recuperate or bounce back accordingly [19,20,21]. Supporting these conceptualisations, research has established evidence for a wide variety of psycho-behavioural benefits related to self-compassion, including building positive attitudes toward oneself [22], establishing achievement goals and greater coping [23], being more prosocial and caring towards others [24], as well as reduced psychological distress and enhanced social safeness during the COVID-19 pandemic [25].

In the context of promoting physical activity and its related health behavioural change, self-compassion likely plays a role despite its novelty. In an investigation of 105 adults who had experienced an exercise setback (e.g., failing to make time for planned exercise, unpleasant experience such as injury) within the last 6 months, Semenchuk et al. found that those high in self-compassion demonstrated greater re-engagement with exercise goals and attenuated negative affect and emotional responses to setbacks [15]. More importantly, these researchers established evidence that increases in self-compassion were associated with greater intrinsic motivation and identified regulation but lowered external regulation and amotivation [15]. They further reasoned and explained the findings, suggesting that self-compassion can help an individual embrace setbacks in exercise, reduce emotional and maladaptive responses, and thus enhance motivation and self-regulation of exercise [26,27]. In support of this view, more recently, Signore et al. [14] replicated Semenchuk et al.’s study in a larger and gender-balanced sample (*n* = 220). The findings support emotional regulation and motivational benefits of self-compassion in those who experienced exercise lapses or setbacks [14]. Interestingly, Signore et al. found that individuals high in masculinity particularly benefit from self-compassion in establishing the identified regulation of exercise and preventing amotivation—probably due to masculinity being a risk factor for emotional dysregulation in sport and exercise contexts and thus more prominently protected by self-compassion [28,29].

While evidence supports the usefulness of self-compassion in the self-regulation of exercise, none of the aforementioned studies tested the relationship between one’s self-compassion and actual physical activity level. Two recent exceptional studies examined the relationship but yielded contradictory findings: one supported a positive association between one’s self-compassion and physical activity behaviour [16], but the other did not [17]. To expand, in 102 Canadian women who were at risk of cardiovascular disease, Semenchuk et al. [16] found those with higher levels of self-compassion achieved greater baseline physical activity levels (accelerometer accessed), fewer emotional responses towards receiving news about their ‘at-risk’ of cardiovascular disease, and more increased physical activity levels at a 3-week follow-up. However, in another study of 169 mid-aged adults in Australia, Hallion et al. [17] found that self-compassion did not account for self-report physical activity behaviour after controlling for barrier self-efficacy (i.e., one’s confidence in overcoming barriers to engage in physical activity; see also [30,31,32,33]).

A major limitation of the two existing studies examining the relationship between self-compassion and physical activity levels is the insufficient statistical power or the lack of sensitivity to detect a true effect or reject a null hypothesis when there is one [34]. Based on the effect size and recruited sample reported in Semenchuk et al. [16] and Hallion et al. [17], posthoc power analysis using G*Power [35] suggests that the former achieved 0.65 power whilst the latter achieved only 0.10 power, representing only 65% and 10% chance, respectively, of detecting a true effect if there is one. Both were below the recommended 0.80 cut-off criterion [36]. Another concern over the limited, contradictory, existing research on self-compassion and physical activity is its lack of consideration of potential mechanistic factors underlying the hypothesised influence. According to the ‘behavioural epidemiology’ framework [37,38], health behaviours such as physical activity are not changed by the ‘intervention’ per se but by its proximal influencers or the so-called mediating variables. Scarce knowledge of what accounts for the influence of self-compassion on physical activity levels makes it difficult to incorporate self-compassion-related practices for promoting physical activity—an increase in self-compassion may not directly increase one’s physical activity levels, but it can contribute to the development of other facilitative factors (e.g., better self-regulation and emotion, enhanced self-efficacy) that promote physical activity subsequently.

**Figure 1:**
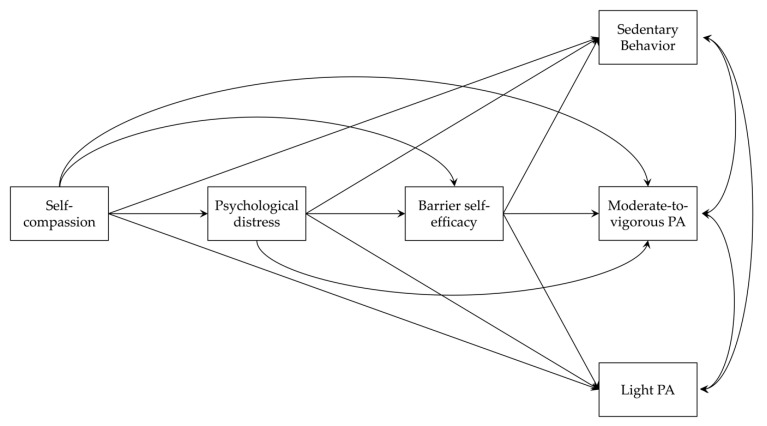
An illustration of the conceptualised multi-mediator multi-outcome model examining the direct and indirect effects of self-compassion on sedentary behaviour, light physical activity (PA), and moderate-to-vigorous PA via reduced psychological distress and increased barrier self-efficacy. Each uni-directional, arrowed path represents a direct conceptual effect, whilst each bi-directional path represents the synchronous correlation of a pair of study outcomes.

To provide a more accurate, less biased insight into the influence of self-compassion in physical activity, the present study aimed to test the association between self-compassion and physical activity in a large, well-powered sample of the general adult population. In light of the emotional [39,40] and coping [23,41] benefits related to self-compassion and the proximal influence of barrier self-efficacy in physical activity behaviour [30,31,42,43], we hypothesised that self-compassion exerts positive influences in one’s physically active health behaviour (i.e., increases physical activity levels and reduces sedentary behaviour) via alleviated psychological distress (thus greater emotion and coping resources; see [44]) and subsequently enhanced barrier self-efficacy (thus greater confidence and motivation; see [32]). Figure 1 delineates the conceptual model we tested in this study. The study will extend the literature [14,15], generate new knowledge of the association between self-compassion and actual physical activity levels, and provide an important, novel understanding of underpinning psycho-behavioural mechanisms of the association between one’s self-compassion and physical activity levels.

## 2. Materials and Methods

### 2.1. Participants

We recruited 569 healthy adults in the UK (Mean age = 41.92 years, SD = 13.70; 47.8% female) to the study. These participants were from different regions in the UK, with the top 5 areas of living being *South East England* (14.8%), *North West England* (12.1%), *Yorkshire and the Humber* (10.9%), *South West England* (10.5%) and *East of England* (8.8%). Most of them were *White British* (89.8%), with the rest being *Asian or Asian British* (3.9%), *Black*, *Caribbean*, *African or Black British* (3.9%), or *Mixed or multiple ethnic groups* (2.3%). A Monte Carlo power analysis for mediation [45] suggested that this sample size allowed us to detect a small indirect effect (i.e., beta coefficients for all paths = 0.11, standardised total indirect effect = 0.02, alpha = 0.05, power = 0.82) of self-compassion on study outcome variables in our hypothesised model (see Figure 1). All participants provided consent prior to taking part in this study.

### 2.2. Measures

#### 2.2.1. Self-Compassion

We employed the 12-item *Self-Compassion Scale—Short* (SCS-S) [46] to assess participants’ dispositional self-compassion. The SCS-S is a short, validated form of the original SCS [47] and has been used successfully in health and behavioural science research [48,49,50]. Participants rated each SCS-S item on a 5-point Likert scale ranging from 1—“almost never” to 5—“almost always”, based on their feelings towards personal failures and distress (e.g., “When I fail something important to me, I become consumed by feelings of inadequacy”). We generated average scores for further analysis, with higher scores reflecting greater self-compassion.

#### 2.2.2. Psychological Distress

We used the 6-item *Psychological Distress Scale* (K-6) [44] to assess participants’ psychological distress. The K-6 is a widely used, quick, and reliable tool for screening and assessing risk for mental illness in the general population [51,52]. It contains six items describing personal feelings or experiences of psychological distress (e.g., “restless or fidgety”, “everything was an effort”, “worthless”). Participants rated K-6 items on a 5-point Likert scale from 0—“none of the time” to 4—“all of the time” based on their experience during the past 30 days. We used K-6 mean scores as an indicator of participants’ level of psychological distress.

#### 2.2.3. Barrier Self-Efficacy

We adopted the 5-item *Self-Efficacy Inventory* (SEI) [53] to assess the extent to which participants were confident of overcoming barriers to engaging in regular physical activity. SEI-based measures manifest excellent internal consistency and have been used widely in physical activity research [31,54,55]. Participants rated their confidence to participate in regular exercise under challenging conditions (e.g., “When it is raining or snowing”) on an 11-point Likert scale ranging from 0—“0% or not confident at all” to 10—“100% or very much confident”. We calculated mean scores for further analysis, with higher scores indicating a higher-level of self-efficacy in overcoming barriers to engaging in physical activity.

#### 2.2.4. Physical Activity

We used the 7-item *International Physical Activity Questionnaire—Short Form* (IPAQ-SF) [56] to assess participants’ physical activity. IPAQ-SF is the short version of the original IPAQ, of which both were developed by a group of physical activity experts for population-level monitoring of physical activity in adults [56]. The IPAQ-based measure is one of the most widely used self-report measures for physical activity and demonstrates good psychometric properties such as high reliability and construct validity [57]. We used the IPAQ-SF to assess participants’ duration and frequency of vigorous, moderate, and light physical activity in the last 7 days. Participants also reported average sitting time on a weekday over the last week. Following the literature [58], we generated average time spent in moderate-to-vigorous physical activity (MVPA; min/day), light physical activity (LPA; min/day), and sedentary behaviours (SB; min/day) for further analysis.

### 2.3. Procedures

With institutional ethics approval (ethics number: ETH2122-2579), we built the study measures together with a set of brief demographic questions into an online, cross-sectional survey using Qualtrics (Provo, UT, USA). To include healthy adult participants in the UK from as wide and diversified backgrounds as possible, we advertised and delivered the online survey via Prolific (i.e., the UK’s largest cloud-sourcing research participation platform; see https://www.prolific.co.uk), where participants were recruited. We used Prolific’s built-in screening function to restrict study recruitment among UK citizens who were over 18 years old and distributed the study as evenly as possible to male and female participants. We posted brief study information and provided an online survey link for interested research participants on Prolific to access the study webpage, where the full study information was presented, followed by a digital consent form. Participants were only allowed to take part in this study once participant consent was provided. On completion of the online survey, we thanked and debriefed participants before directing them back to Prolific with an automatically generated code to log their participation in this study. Participants who successfully completed the survey within a reasonable timeframe (i.e., within 3 standard deviations of the mean time spent on completing the survey, thus not being too quick or staying too long in the online survey) received a £1 incentive via Prolific. The incentive rate was based on the minimum Prolific rate and estimated survey completion time.

### 2.4. Data Analysis

We first checked missing data, descriptive statistics, and Pearson correlations between study variables in IBM SPSS Version 28. Following the preliminary analysis, Mplus Version 8 was used to test the hypothesised serial mediation model [59] (see Figure 1). We focused on examining the direct and indirect effects of self-compassion on average daily time spent on MVPA, LPA, and SB, whilst testing psychological distress and barrier self-efficacy as the underpinning, mediating factors. We also examined the direct and indirect effects of self-compassion on barrier self-efficacy, with psychological distress being the hypothesised mediator. When testing the hypothesised model, we adjusted demographic differences in age and gender for study outcome variables (i.e., MVPA, LPA, and SB) and used *Full Information Maximum Likelihood* (FIML) approach to address missing data for more accurate estimation and greater statistical power [60]. The bootstrapping method using 10,000 resamplings was used when testing the model, as such an approach provides more accurate standard errors for parameter estimations and alleviates impacts of potential data non-normality and sampling error [61]. Following recommendations from Hu and Bentler [62], we used Chi-square (*χ*^2^), comparative fit index (CFI), and standardised root mean square residual (SRMR) tests to assess model fit, with larger than or close to 0.95 CFI, smaller than or close to 0.08 SRMR indicating a good model fit. For standardised estimates on direct (*β*) and indirect effects, the precise *p*-value (significant at 0.05), and 95% bootstrapping confidence interval (CI) of the estimates were reported. We interpreted any effect with 95% CI bounded but not encompassing zero as marginal despite a greater than 0.05 *p*-value. 

## 3. Results

### 3.1. Preliminary Analysis

No missing data were found in key study variables (see Section 2.2). Skewness and kurtosis were within ±1.76 and ±3.56, respectively, for all study variables. This fulfilled the recommended criteria of ±3 for skewness and ±10 for kurtosis for running path models [63]. Cronbach’s alpha was 0.82, 0.90, and 0.89 for self-compassion, psychological distress, and barrier self-efficacy measure, respectively, suggesting the very good to excellent internal consistency of study measures. As was predicted, self-compassion manifested a strong negative correlation with psychological distress, a moderate positive correlation with barrier self-efficacy, and a small positive correlation with average daily time spent in MVPA. Psychological distress manifested a moderate negative correlation with barrier self-efficacy and a small negative correlation with MVPA. Barrier self-efficacy manifested a small, negative correlation with average daily sedentary time, a small, positive correlation with time spent engaging in light PA, and a moderate-to-large correlation with MVPA. Table 1 displays the descriptive statistics, Cronbach’s alpha (if applicable), and correlations of all study variables.

### 3.2. Main Analysis

Test of the serial mediation model yielded good model fit: *χ*^2^ = 36.15, *df* = 1, *p* = 0.00; CFI = 0.95, SRMR = 0.04. Figure 1 illustrates the model diagram. Table 1 and Table 2 display all regression statistics for each direct and indirect path tested in the model, respectively.

#### 3.2.1. Direct and Indirect Effects on Moderate-to-Vigorous Physical Activity (MVPA)

The model accounted for a 15.8% variance in MVPA. Barrier self-efficacy was directly related to the increased time spent in MVPA (*β* = 0.40, *p* = 0.00, 95% CI [0.31, 0.48]), but not self-compassion (*β* = 0.01, *p* = 0.84, 95% CI [−0.10, 0.12]) nor psychological distress (*β* = 0.00, *p* = 0.97, 95% CI [−0.12, 0.12]). Importantly, self-compassion demonstrated a significant, positive indirect effect on MVPA via increased barrier self-efficacy (*β* = 0.15, *p* = 0.01, 95% CI [0.04, 0.27]; standardised indirect effect = 0.06, *p* = 0.01, 95% CI [0.01, 0.11]). Moreover, the serial mediation path of self-compassion on MVPA via decreased psychological distress (*β* = −0.75, *p* = 0.00, 95% CI [−0.78, −0.71]) and subsequently increased barrier self-efficacy (*β* = −0.11, *p* = 0.06, 95% CI [−0.23, 0.01]) was marginal and positive (standardised indirect effect = 0.03, *p* = 0.07, 95% CI [0.00, 0.07]). These findings suggested that individuals high in self-compassion spent more time in MVPA due to attenuated psychological distress and increased barrier self-efficacy. See Table 2 and Table 3 for all statistic details.

#### 3.2.2. Direct and Indirect Effects on Light Physical Activity (LPA)

The model accounted for a 1.5% variance in LPA. Barrier self-efficacy was directly related to the increased time spent engaged in LPA (*β* = 0.13, *p* = 0.02, 95% CI [0.02, 0.23]), but not self-compassion (*β* = −0.04, *p* = 0.58, 95% CI [−0.17, 0.09]) nor psychological distress (*β* = −0.02, *p* = 0.81, 95% CI [−0.14, 0.11]). Nevertheless, self-compassion demonstrated a marginal, positive indirect effect on LPA via increased barrier self-efficacy (standardised indirect effect = 0.02, *p* = 0.08, 95% CI [0.00, 0.04]). Furthermore, the serial mediation path of self-compassion on LPA via decreased psychological distress and subsequently increased barrier self-efficacy was also marginal and positive (standardised indirect effect = 0.01, *p* = 0.10, 95% CI [0.00, 0.03]). These findings provide partial support to self-compassion’s indirect effect on increasing LPA via attenuated psychological distress and increased barrier self-efficacy. See Table 2 and Table 3 for all statistic details.

#### 3.2.3. Direct and Indirect Effects on Sedentary Behaviour (SB)

The model accounted for a 1.1% variance in SB. Barrier self-efficacy was directly related to decreased time spent in SB (*β* = −0.09, *p* = 0.03, 95% CI [0.02, 0.23]), but not self-compassion (*β* = −0.06, *p* = 0.36, 95% CI [−0.17, 0.09]) nor psychological distress (*β* = −0.05, *p* = 0.45, 95% CI [−0.14, 0.11]). Nevertheless, self-compassion demonstrated a marginal, negative indirect effect on SB via increased barrier self-efficacy (standardised indirect effect = −0.01, *p* = 0.10, 95% CI [−0.03, 0.00]). In addition, the serial mediation path of self-compassion on SB via decreased psychological distress and subsequently increased barrier self-efficacy was also marginal and negative (standardised indirect effect = −0.01, *p* = 0.10, 95% CI [−0.02, 0.00]). These findings provide partial support for self-compassion’s effect on reducing SB. See Table 2 and Table 3 for all statistic details.

#### 3.2.4. Direct and Indirect Effects on Barrier Self-Efficacy (BSE)

The model accounted for a 6.2% variance in BSE. Self-compassion was directly related to greater BSE (*β* = 0.15, *p* = 0.01, 95% CI [0.04, 0.27]). Psychological distress’s direct effect on BSE was negative but marginal (*β* = −0.11, *p* = 0.06, 95% CI [−0.23, 0.00]). Importantly, the indirect effect of self-compassion on barrier self-efficacy via decreased psychological distress (*β* = −0.75, *p* = 0.00, 95% CI [−0.78, −0.71]) received marginal support (standardised indirect effect = 0.08, *p* = 0.10, 95% CI [0.00, 0.17]). These findings reveal that self-compassion manifests meaningful direct and indirect effects contributing to enhanced barrier self-efficacy for physical activity. See Table 2 and Table 3 for all statistic details. 

## 4. Discussion

### 4.1. Summary of Findings

To the best of our knowledge, this is the first well-powered study investigating the relationship between self-compassion and physical activity behaviour and testing the mediating factors underlying this relationship. Using a sample of 569 healthy adults in the UK, we tested a multi-mediator multi-outcome model (see Figure 1) and examined the hypothesis that self-compassion links to higher-level physical activity via mitigated psychological distress and subsequently enhanced barrier self-efficacy. Our data revealed that individuals high in self-compassion appeared to engage in greater levels of physical activity, of which the positive association was underpinned by reduced psychological distress and increased barrier self-efficacy. The observed direct and indirect effects of self-compassion were stronger when examining more vigorous physical activity (i.e., MVPA) in comparison to less vigorous physical activity (i.e., light PA) and sedentary behaviour. Self-compassion appeared to be a source for building barrier self-efficacy, thanks to its emotional benefits and associated lower levels of psychological distress. The findings reveal the usefulness of self-compassion in promoting physical activity, especially in overcoming barriers to engaging in regular physical activity. This is important as deliverers and health professionals work with people when facilitating physical activity.

### 4.2. Highlights and Implications

While our test of the hypothesis was robust and the findings are clear, we highlight several noteworthy points for researchers, practitioners, and other stakeholders, when considering embedding self-compassion for the promotion of physical activity. First, self-compassion appears to be more influential to more MVPA compared to low-intensity activity or sedentary behaviour. This was supported by our data that the indirect effect of self-compassion was greater on MVPA than that on light physical activity and sedentary behaviour. A possible explanation is that self-compassion helps an individual to overcome barriers and recuperate from exercise-related setbacks [14,15], and one may be more susceptible to challenges, barriers, or potential setbacks when engaging in more vigorous physical activity (e.g., experiencing fatigue, difficulty in generating motivation, injury). Also, one may be more susceptible to challenges when motivating oneself to start and complete a challenging, vigorous PA episode or session, and adopting a self-compassionate mind may allow one to accept these challenges with a gentler mind, thus enhancing the capacity to overcome them.

The findings also align with existing literature that factors contributing to MVPA may not necessarily benefit equally from less vigorous physical activity [31]. Alternatively, since our data suggested that the influence of self-compassion on physical activity is more indirect via other underpinning factors (e.g., psychological distress, barrier-efficacy) rather than direct, it is possible the benefit of self-compassion in promoting light physical activity was underestimated in this study, due to the missing of other potential underlying factors. Nevertheless, given growing evidence of health benefits linked to less vigorous types of physical activity [64,65,66], one would do well to consider and examine the usefulness of self-compassion in promoting light physical activity, which may be particularly beneficial to those who are more vulnerable than others, or who have a disability and struggle to engage in greater than light physical activity. Also, one should be aware that what is considered light for one person may be moderate or vigorous for others, such as frail elderly or those new to physical activity and low in fitness. Therefore, self-compassion could be more beneficial than expected in certain populations.

Furthermore, among the tested influencers (i.e., self-compassion, psychological distress, barrier self-efficacy, and demographic differences) of physical activity behaviour (regardless of intensity), barrier self-efficacy accounted for the most significant proportion of variance in one’s physical activity. Meanwhile, barrier self-efficacy underpinned the positive influence of self-compassion and the negative influence of psychological distress in one’s physical activity. This finding is comparable to existing literature that barrier self-efficacy is a more proximal driver of physical activity [33] and underlies the positive influences of task self-efficacy and perceived enjoyment PA on physical activity levels [31]. Also, a recent systematic review [67] has demonstrated inconclusive evidence of mindfulness training in increasing physical activity, probably due to a lack of consideration of mechanistic psycho-behavioural factors in the design and implementation of interventions (see [68] for a comparison between mindfulness and self-compassion). This again calls for attention to the importance of tackling factors underpinning physical activity when developing and delivering an intervention. Building on existing knowledge of establishing barrier self-efficacy (e.g., promoting enjoyment and task self-efficacy, see [31]), the current study adds new insight into how self-compassion can be a source for greater self-efficacy to overcoming barriers in physical activity. Future self-efficacy-based physical activity interventions would do well to tackle barrier self-efficacy [54,55,69] and could also consider self-compassion as an important psychological resource for individuals to overcome challenges and difficulties when engaging in physical activity (see [70] for a pilot clinical trial of self-compassion intervention promoting physical activity in people with prediabetes).

Last but not least, despite being a trait-like dispositional characteristic, an individual can adopt greater self-compassion via purposely designed compassionate mind training (for a review, see [71]). However, one should note that most existing compassionate mind training is not immediately transferrable to facilitate physical activity behavioural changes, because they are more therapy-centred [72]. As such, to optimise the use of self-compassion in facilitating adaptation and maintenance of physical activity, researchers and practitioners would do well to design and implement specific compassionate mind training that is tailored to support physical activity (e.g., for enhanced self-regulation of exercise to facilitate overcoming challenges and barriers). There is also a need for PA-specific compassionate mind training to include training for the promoters (i.e., those who deliver the intervention or programme). This would require appropriate, evidence-based dissemination of the knowledge (e.g., the role of a self-compassionate mind in physical activity) as well as cautious design and development of contents for both the intervention promoters and participants.

### 4.3. Limitations, Strengths and Other Future Directions

The current study is not without limitations. One noticeable weakness is the cross-sectional design of the study, which does not allow us to evaluate the potential causal effect of self-compassion on physical activity. However, the study also features several important strengths. Given the novelty of the study, its well-powered sample and the examination of proximal influencers underpinning self-compassion and physical activity, the cross-sectional design should not undermine the value of our findings in understanding the association between self-compassion and physical activity and the underlying factors that may explain this relationship. Importantly, the present study opens the directions for future studies that could further investigate longitudinal, causal effects of self-compassion on one’s physical activity and offers valuable hints in developing compassion-focused interventions for exercise promotion. Physical activity providers would also benefit from the new knowledge generated from this study when considering the design and delivery of future PA interventions.

Besides, one may have concerns over the use of self-report measures (i.e., IPAQ) to assess participants’ physical activity levels. Indeed, a comparison of self-report physical activity measures and accelerometer-based PA [73] suggested that the IPAQ is prone to recall bias and tends to overestimate actual time spent in physical activity. While fully acknowledging the shortcomings of self-report physical activity such as the IPAQ, we assure that physical activity levels assessed via the IPAQ in this study were not interpreted as the actual, absolute levels of physical activity. Instead, we used physical activity data assessed by the IPAQ for a relative comparison among study participants, which was the original purpose of the IPAQ and has been proven reliable when examining physical activity levels in multiple countries [56,57,74]. Future research would do well to examine the replication of findings from the present study using accelerometer-based assessment.

Finally, although this study focused on examining psychological distress and barrier self-efficacy as the serial mediating factors underpinning the relationship between self-compassion and physical activity, it is possible other psychosocial-behavioural factors or physiological factors, such as increased dopamine levels, may underly the observed effect of self-compassion on physical activity (see [75] for a review of dopamine and physical activity and [76] for a review of a neurobiological link between compassion and dopamine). Future research should consider testing alternative mechanistic factors, thus understanding more fully how self-compassion can be built into the design and delivery of interventions so as to promote physical activity.

## 5. Conclusions

Unlike other personality traits or dispositions, self-compassion can be nurtured and has the potential to positively influence how one engages in physical activity. Using a well-powered adult sample from the UK, we provided the first evidence that self-compassion’s positive influence on physical activity operates through decreased psychological distress and subsequently increased barrier self-efficacy. The findings suggest that self-compassion is a good source of emotional resources (i.e., attenuated psychological distress) and confidence to overcome challenges and obstacles (i.e., increased barrier self-efficacy) in the context of physical activity. Self-compassion contributes more strongly to moderate and vigorous physical activity levels compared to lower-intensity activity and sedentary behaviour. We call for attention to the potential use of self-compassion in promoting physical activity adoption and maintenance.

## Figures and Tables

**Table 1:** Descriptive statistics and zero-order correlations for the study variables.

Measure	1	2	3	4	5	6	7	8
1. Age (year)		0.24 **	0.20 **	−0.32 **	0.05	−0.08 *	−0.04	−0.04
2. Sex (0—female, 1—male)		–	0.11 **	−0.19 **	0.07	−0.05	0.01	0.11 **
3. Self-compassion			(0.82)	−0.75 **	0.25 **	−0.05	0.01	0.12 **
4. Psychological distress				(0.90)	−0.23 **	0.03	−0.02	−0.11 **
5. Barrier self-efficacy					(0.89)	−0.10 *	0.13 **	0.39 **
6. Sedentary time (min/day)							−0.18 **	−0.10 *
7. LPA (min/day)								0.18 *
8. MVPA PA (min/day)								
**Mean**	41.92	0.52	3.26	2.46	5.17	421.74	40.53	29.26
**SD**	13.70	0.50	0.84	0.99	2.55	210.41	36.38	27.42

*Note*. PA = physical activity, LPA = light physical activity, MVPA = moderate-to-vigorous physical activity, SD = standard deviation. Numbers in parenthesis represent Cronbach’s alpha as an indicator of internal consistency of the target measure. * *p* < 0.05, ** *p* < 0.01.

**Table 2:** Unstandardised and standardised direct effect of hypothesised paths.

Variable	SC	PD	BSE	SB	LPA	MVPA
SC		−0.85 (−0.75) *	0.47 (0.15) *	−14.95 (−0.06)	−1.58 (−0.04)	0.38 (0.01)
PD			−0.30 (−0.11)	−11.23 (−0.05)	−0.58 (−0.02)	0.06 (0.00)
BSE				−7.61 (−0.05) *	1.78 (0.13) *	4.25 (0.40) *

*Note*. SC = self-compassion, PD = psychological distress, BSE = barrier self-efficacy, SB = sedentary behaviour (min/day), LPA = light physical activity (min/day), MVPA = moderate-to-vigorous physical activity (min/day). Unstandardised and standardised estimates are displayed without and with parentheses, respectively. Unstandardised estimates of direct effect can be interpreted as the averaged raw change of outcome variable per 1 unit change in predicting variable. * indicates significant effect.

**Table 3:** Unstandardised and standardised indirect effect of hypothesised paths.

Mediation Path	Indirect Effect	Bootstrap SE	Bootstrap 95% CI
SC→BSE→SB ^	−3.55 (−0.01)	2.22 (0.01)	[−7.91, 0.81] (−0.03, 0.00)
SC→PD→SB	9.61 (0.04)	12.71 (0.05)	[−15.31, 34.52] (−0.06, 0.14)
SC→PD→BSE→SB ^	−1.93 (−0.01)	1.50 (0.01)	[−4.87, 1.00] (−0.02, 0.00)
SC→BSE→LPA ^	0.83 (0.02)	0.48 (0.01)	[−0.12, 1.77] (0.00, 0.04)
SC→PD→LPA	0.49 (0.01)	2.00 (0.05)	[−3.43, 4.42] (−0.08, 0.10)
SC→PD→BSE→LPA ^	0.45 (0.01)	0.32 (0.01)	[−0.18, 1.08] (0.00, 0.03)
SC→BSE→MVPA *	1.98 (0.06)	0.78 (0.02)	[0.45, 3.51] (0.01, 0.11)
SC→PD→MVPA	−0.05 (−0.00)	1.49 (0.05)	[−2.96, 2.86] (−0.10, 0.09)
SC→PD→BSE→MVPA ^	1.08 (0.03)	0.60 (0.02)	[−0.10, 2.26] (0.00, 0.07)
SC→PD→BSE ^	0.25 (0.08)	0.14 (0.04)	[0.00, 0.52] (0.00, 0.17)

*Note*. SE = standard error, CI = confidence interval, SC = self-compassion, PD = psychological distress, BSE = barrier self-efficacy, SB = sedentary behaviour (min/day), LPA = light physical activity (min/day), MVPA = moderate-to-vigorous physical activity (min/day). Unstandardised and standardised estimates are displayed without and with parentheses, respectively. Unstandardised estimates of indirect effect can be interpreted as the averaged raw change of outcome variable per 1 unit change in predicting variable via its associated change in the mediating variable(s). ^ indicates meaningful, marginal effect, * indicates significant effect.

## Data Availability

The data are not publicly available due to ethics restrictions. The data can be obtained from the corresponding author based on reasonable request for research purposes.
